# Visual performance, safety, and patient satisfaction after binocular clear lens extraction and trifocal intraocular lens implantation in Chinese presbyopic patients

**DOI:** 10.1186/s12886-024-03573-9

**Published:** 2024-07-23

**Authors:** Lulu Chen, Lu Sun, Yongxiang Tang, Wenda Sui, Ailing Bian, Xia Zhang, Zaowen Wang, Yong Zhong, Shunhua Zhang

**Affiliations:** 1 Department of Ophthalmology, Peking Union Medical College Hospital, Chinese Academy of Medical Sciences, No.1, Shuaifuyuan Wangfujing Dongcheng District, Beijing, 100730 China; 2 Department of Ophthalmology, The Affiliated Hospital of Zunyi Medical University, Zunyi, 563000 Guizhou China

**Keywords:** Presbyopia, Refractive lens exchange, Trifocal intraocular lens, Visual performance, Patient satisfaction

## Abstract

**Background:**

Addressing presbyopia in the aging population, particularly in non-cataractous patients, remains a challenge. This study evaluates the outcomes of refractive lens exchange (RLE) with AT LISA tri 839MP trifocal intraocular lens (IOL) implantation in a Chinese presbyopic population without cataracts.

**Methods:**

The study included 164 eyes from 82 patients undergoing bilateral RLE at Peking Union Medical College Hospital. Comprehensive evaluations encompassed visual acuities, refraction, ocular aberrometry, and subjective outcomes via the VF-14 questionnaire. The focus was on postoperative visual performance, refractive outcomes, safety, objective optical quality, and patient satisfaction.

**Results:**

100%, 90.2%, and 89.0% of patients achieved binocular UDVA, UNVA, and UIVA of logMAR 0.1 or better at 6 months postoperatively. 97.6% of eyes were within ± 1.00 D of emmetropia postoperatively. Optical quality assessments showed increases in modulation transfer function and Strehl ratios (*p* < 0.05). High-order aberrations decreased significantly (*p* < 0.05). Despite the high incidence of posterior capsule opacification (83.2%), managed with early Nd: YAG capsulotomy, no other severe complications were reported. Patient-reported outcomes indicated high satisfaction, with an average VF-14 score of 94.3 ± 10.2 and 93.5% achieving complete spectacle independence. Halo (66.2%) was the most commonly reported optical phenomena, followed by glare (18.2%), and starburst (7.8%) after surgery.

**Conclusions:**

Bilateral RLE with trifocal IOLs in presbyopic patients without cataracts significantly improves visual acuity and reduces ocular aberrations in presbyopic patients. The procedure offers high patient satisfaction and spectacle independence, though it requires careful patient selection and management of expectations regarding potential photic phenomena.

## Background

Presbyopia constitutes the primary cause of near vision impairment beyond the fourth decade of life [1–3], with profound implications for self-perception, life satisfaction, work performance, and social interaction. This irreversible condition, stemming partly from alterations in the viscoelastic properties of the crystalline lens, was estimated to affect 510 million individuals globally in 2020, a number projected to reach 866 million by 2050 [1]. Contemporary presbyopia treatment strategies span from noninvasive modalities, such as spectacles, to more enduring interventions including laser refractive correction, scleral procedures, phakic intraocular lens (IOL) implantation including implantable collamer lens with EDOF features, and clear lens extraction with subsequent IOL implantation [4–7]. Among these, refractive lens exchange (RLE) is garnering attention for its dual ability to correct presbyopia and prevent cataracts. Distinguished from cataract surgery by its elective nature in patients without significant lens opacity, RLE is increasingly preferred by those seeking independence from corrective lenses [8–10].

Although RLE and cataract surgery share procedural similarities, they serve distinct patient populations. RLE patients typically present with higher visual expectations, attributed to the initial clarity of their lenses [11]. These patients seek not only visual acuity but also high-quality vision free from disturbances such as glare and halos [12]. RLE demands advanced technical proficiency, and there is a need for a nuanced appreciation of the associated risks. Within China, RLE is not widely adopted, with many ophthalmologists adopting a cautious or adverse stance toward the procedure. Thus, a thorough understanding of the risk–benefit profile specific to the Chinese demographic is essential.

Research documenting the outcomes of RLE remains scarce, particularly for Chinese populations. This study was designed to enhance the clinical dataset and expand the understanding of RLE by reporting on visual performance, objective optical quality, patient-reported outcomes, and the safety profile of RLE with trifocal IOL implantation in a cohort of Chinese presbyopic patients without cataracts.

## Methods

### Patients

This retrospective analysis encompassed individuals who underwent bilateral RLE and IOL implantation at Peking Union Medical College Hospital between January 2021 and March 2023. The AT LISA 839MP IOLs, manufactured by Carl Zeiss Meditec in Germany, were utilized for all participants. The conduct of the study was in strict accordance with the principles of the Declaration of Helsinki and received approval from the institutional review board of PUMCH (I-22PJ782). Informed consent was obtained from all participants.

Participants expressed a definitive preference for multifocal IOLs to address their refractive errors and presbyopia. Inclusion criteria consisted of individuals with bilateral presbyopia absent of cataracts, fulfilling the binocular requisites for trifocal IOLs. Presbyopia was characterized by at least 2.00 D add to the best optical distance correction to achieve near visual acuity of J1. A 'clear lens' was defined as one with clinically negligible lens opacity and a preoperative best-corrected visual acuity (BCVA) of 0.0 LogMAR or better. Criteria for trifocal IOL candidacy included regular corneal astigmatism under 1.5 D (with the rule) or 1.0 D (against the rule) and a scotopic pupil diameter of less than 6.0 mm. Exclusion criteria encompassed a history of ocular conditions (e.g., keratoconus, pseudoexfoliation syndrome, uveitis, glaucoma, diabetic retinopathy, pathologic myopia, and trauma), prior ocular surgeries (e.g., keratorefractive surgery, vitrectomy, and scleral buckling), atypical corneal higher-order aberrations, and a scotopic pupil size exceeding 6 mm.

### Preoperative evaluation

Preoperative assessments for each patient encompassed a comprehensive ophthalmic evaluation, which included manifest refraction, monocular and binocular uncorrected visual acuities at distance (UDVA), intermediate (UIVA) at 80 cm, and near (UNVA) at 40 cm, as well as corrected distance visual acuity (CDVA). Additional examinations included intraocular pressure measurement, slit-lamp examination, corneal topography utilizing the Pentacam system (Oculus Optikgeräte, Wetzlar, Germany), endothelial cell count using the SP-2000 (Topcon, Japan), dilated fundus examination, B-scan ultrasonography (Compact touch, Lumibird Medical, France) wide-field fundus photography (Optos PLC, Dunfermline, United Kingdom), and optical coherence tomography (OCT) of the macula (DRI-1; Topcon, Japan).

### Optical biometry

Optical biometry measurements were conducted using the IOL Master 700 (Carl Zeiss Meditec, Germany), with intraocular lens (IOL) calculations based on the Barrett Universal II TK formula. The target refraction was set to approximate emmetropia.

### Ocular aberrometry

For ocular aberrometry, the iTrace aberrometer (Tracy Technologies, Houston, USA) was employed. This device combines ray tracing aberrometry with corneal topography to derive comprehensive aberration data for visual quality analysis. Measurements were performed under scotopic conditions, standardized to a 3 mm pupil size without refractive correction. Preoperative assessments included angle alpha, defined as the radial distance between the limbus center and the visual axis, and angle kappa, indicating the radial distance between the pupil center and the visual axis. The Dysfunctional Lens Index (DLI) quantified age-related changes in the crystalline lens [13]. The modulation transfer function (MTF) curve was developed with the built-in software and the average height of total, corneal, and intraocular MTF values were used to quantify MTF curves. Total, corneal and intraocular Strehl ratios (SR) were calculated to evaluate objective optical quality. Total, corneal, and intraocular aberrations (root mean square, RMS 3 mm) were calculated with the built-in software.

### Surgical procedure

Before the surgical procedure, patients were administered topical anesthetic and mydriatic agents. In cases of corneal astigmatism less than 1 diopter (D), a 2.4 mm limbal incision was created on the steepest corneal meridian. For astigmatism levels ranging from 1D to 1.5D, the same size limbal incision was positioned on the steepest meridian, complemented by an astigmatic keratotomy on the opposing meridian. This was followed by a 5.0 mm continuous curvilinear capsulorhexis (CCC) and standard phacoemulsification. The intraocular lens (IOL) was implanted into the capsular bag using an injector (BLUEMIX 180, Carl Zeiss Meditec) and carefully aligned, ensuring the rhexis margin uniformly overlapped the IOL's edge. Intraoperative complications, if any, were meticulously documented. Postoperatively, all patients were treated with glucocorticoid and antibiotic eye drops for two weeks. The procedure for the second eye was conducted within one week following the initial surgery.

### Objective postoperative evaluation

Patients underwent postoperative assessments at one day, one month, and six months or beyond following the surgical procedure. Objective evaluations included monocular and binocular uncorrected distance visual acuity (UDVA), uncorrected intermediate visual acuity (UIVA) at 80 cm, uncorrected near visual acuity (UNVA) at 40 cm, corrected distance visual acuity (CDVA), corrective intermediate visual acuity (CIVA), and corrected near visual acuity (CNVA). Additional assessments were subjective refraction, anterior and posterior segment examination via slit-lamp, intraocular pressure measurement, and ocular aberrometry, with postoperative aberrometry conducted under identical conditions to the preoperative evaluation. The integrated software facilitated the calculation of average total, corneal, and intraocular MTF, SR values, and aberrations, quantified as the RMS over a 3 mm pupil diameter. Monocular and binocular defocus curves were evaluated 6 months after surgery using the ETDRS chart at 4 m and adding to the patient's manifest refraction + 1.00 to -4.00 D sphere in 0.50 D increments.

### Patient-reported outcomes

On the last clinical visit, patients completed a verified Chinese version of the visual function-14 (VF-14) questionnaire face to face by a trained researcher in order to evaluate subjective satisfaction with the surgery outcome. A scale of 0–100 was used, in which a score of 100 indicates ‘no difficulty ‘, 75 indicates ‘a little difficult’, 50 reflects ‘ moderate difficulty’, 25 means ‘a great deal of difficult’, and 0 reflects ‘ unable to do’. The original VF-14 questionnaire was modified with additional questions reporting the existence and severity of glare, halo, starburst, diplopia; patient satisfaction; and whether they would recommend the surgery to their family or friends. A scale of 1–5 was used to determine the severity of visual disturbance if the patients answered ‘yes’ to any of the visual disturbances. A scale of 1 means ‘slight discomfort’, and a scale of 5 means ‘very annoying’.

### Safety

Intraoperative complications, such as posterior capsule rupture, iris injury, and corneal injury, as well as postoperative complications, including corneal edema, elevated intraocular pressure, and cystoid macular edema, were meticulously documented. Instances of posterior capsule opacification (PCO) were identified, with neodymium-doped yttrium aluminum garnet (Nd: YAG) laser treatment administered in cases where PCO precipitated visual symptoms or resulted in a reduction of UDVA by one or more lines on the ETDRS chart. The frequency of Nd: YAG laser interventions was recorded six months post-surgery.

### Statistical analysis

Statistical analyses were conducted using SPSS version 25.0 (IBM, Chicago, United States). Categorical data underwent chi-square or Fisher's exact test evaluation. The Kolmogorov–Smirnov test assessed the normality of the datasets. Continuous variables are presented as means ± standard deviations. For paired comparisons, the normality of differences was determined using the Kolmogorov–Smirnov test. Depending on normality, paired Student's t-tests or Wilcoxon rank-sum tests compared preoperative to postoperative values. Correlations between variables were analyzed using Pearson or Spearman coefficients, based on normality assumptions. A *p*-value of less than 0.05 denoted statistical significance.

## Results

### Patient characteristics

A total of 164 eyes from 82 subjects (28 males, and 54 females) participated in the study. The cohort's average age was 54.8 ± 6.0 years. The mean axial length was 24.2 ± 2.0 mm, with 74.4% of eyes within the 22.0 mm to 26.0 mm range. The average DLI registered at 7.71 ± 2.53. Preoperative monocular CDVA averaged -0.00 ± 0.05logMAR (range: -0.18 to 0.22), while binocular CDVA averaged -0.03 ± 0.05logMAR (range: -0.18 to 0.10). Preoperative patient characteristics and IOL data are summarized in Table 1.

**Table 1:** Preoperative characteristics and IOL data of the patients

Patient characteristics	Mean ± SD	Range
Age (years)	54.8 ± 6.0	(42.0, 72.0)
Gender (male/female)	28(34.1%) / 54(65.9%)	
Axial length (mm)	24.2 ± 2.0	(20.8, 31.0)
AL < 22.0 mm /22.0 mm ≤ AL < 26.0 mm/ AL > 26.0 mm	8(4.9%)/ 122(74.4%)/ 34(20.7%)	
Anterior chamber depth (mm)	3.2 ± 0.4	(2.4, 4.4)
WTW (mm)	11.9 ± 0.4	(11.0, 13.0)
K1	43.5 ± 1.40	(39.0, 47.7)
K2	44.1 ± 1.5	(39.2, 48.1)
Corneal astigmatism (D)	0.7 ± 0.4	(0.0, 1.7)
IOL power (D)	18.5 ± 5.8	(0.0, 30.5)
Expected spherical equivalent (D)	0.02 ± 0.17	(-0.63, 0.46)
Angle alpha (mm)	0.33 ± 0.14	(0.01, 0.62)
Angle kappa (mm)	0.25 ± 0.13	(0.01, 0.62)
DLI	7.71 ± 2.53	(1.47, 10.00)
monocular UDVA	0.39 ± 0.46	(-0.18, 2.00)
binocular UDVA	0.32 ± 0.44	(-0.18, 1.00)
Monocular UIVA	0.39 ± 0.30	(0.00, 1.30)
Binocular UIVA	0.33 ± 0.30	(0.00, 1.00)
Monocular UNVA	0.52 ± 0.23	(0.00, 1.10)

*Abbreviations: AL* Axial length, *WTW* White to white, *IOL* Intraocular lens, *DLI* Dysfunction lens index, *CDVA* Corrected distance visual acuity, *UDVA* Uncorrected distance visual acuity, *UIVA* uncorrected intermediate visual acuity, *UNVA* Uncorrected near visual acuity, *D* Dioptor

### Visual acuity and defocus curve

**Fig. 1:**
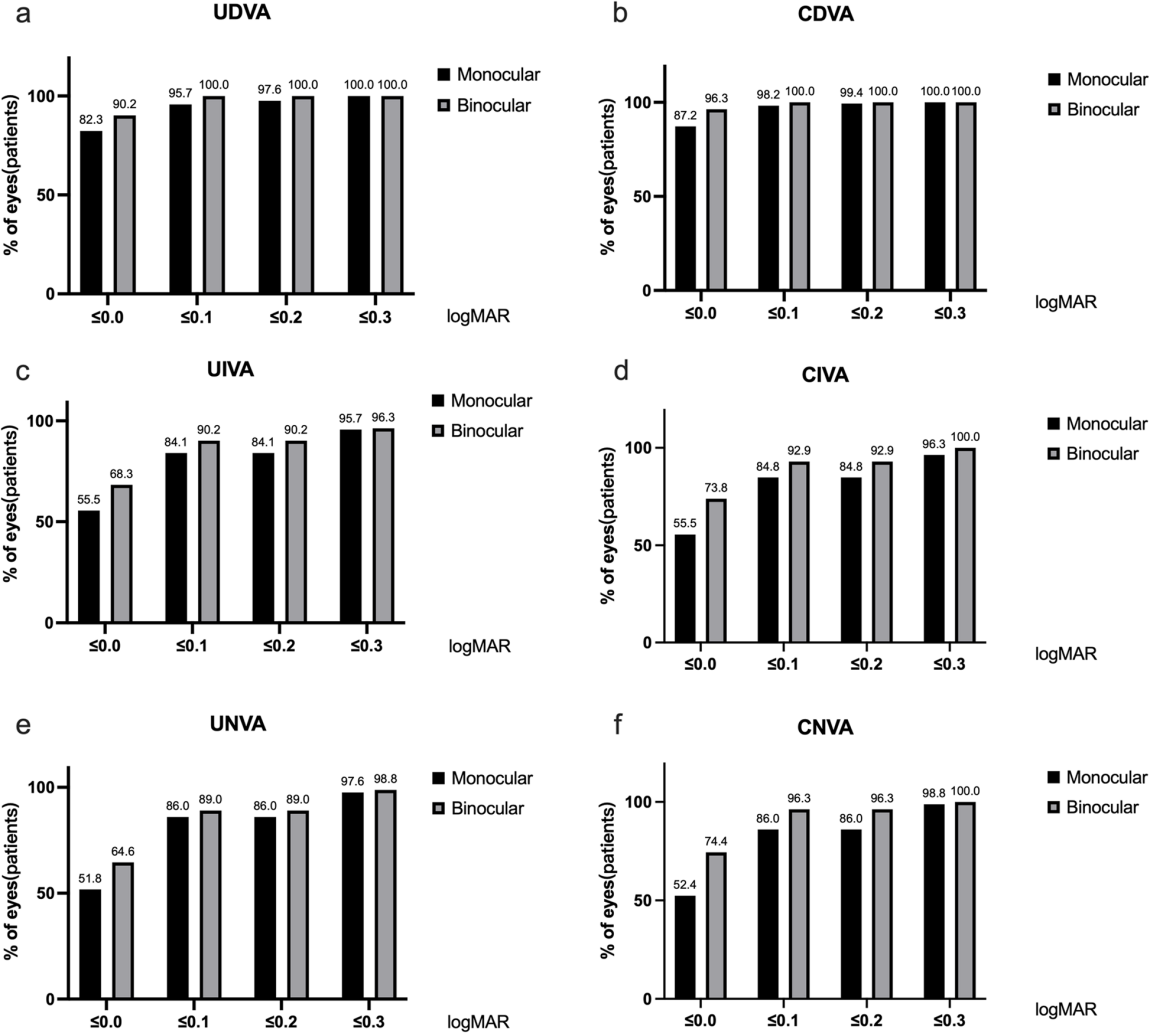
Monocular and binocular cumulative uncorrected (**a**) and corrected distance visual acuity (**b**) at 6 months postoperatively. Monocular and binocular cumulative uncorrected (**c**) and corrected intermediate visual acuity (**d**) at 6 months postoperatively. Monocular and binocular cumulative uncorrected (**e**) and corrected near visual acuity (**f**) at 6 months postoperatively

Comparative analysis between preoperative and postoperative CDVA revealed significant enhancements in both monocular (*p* < 0.001) and binocular (*p* = 0.057) measurements. Improvements in monocular and binocular UDVA, UIVA, and UNVA were significant one-day post-surgery (*p* < 0.001), as documented in Table 2. At the six-month mark, 82.3% of eyes attained a monocular UDVA of 0.0logMAR or better, with 90.2% of subjects achieving similar binocular UDVA benchmarks. Similarly, monocular CDVA improvements were noted in 87.2% of eyes, and binocular CDVA in 96.3% of subjects. For intermediate and near visual acuities, most patients achieve or surpass the 0.1 logMAR threshold (Fig. 1).

**Table 2:** Postoperative visual acuity in logMAR

Visual Acuity(logMAR)	1 day postoperative		1 month postoperative		6 months postoperative	
	**mean ± SD**	**range**	**mean ± SD**	**range**	**mean ± SD**	**range**
Monocular CDVA	-0.03 ± 0.08	(-0.18, 0.30)	-0.01 ± 0.08	(-0.18, 0.40)	-0.03 ± 0.07	(-0.18, 0.22)
Binocular CDVA	-0.06 ± 0.06	(-0.18, 0.10)	-0.04 ± 0.07	(-0.18, 0.30)	-0.05 ± 0.06	(-0.18, 0.10)
Monocular UDVA	-0.02 ± 0.10	(-0.18, 0.30)	0.00 ± 0.10	(-0.18, 0.40)	-0.02 ± 0.08	(-0.18, 0.22)
Binocular UDVA	-0.05 ± 0.08	(-0.18, 0.15)	-0.02 ± 0.10	(-0.18, 0.40)	-0.04 ± 0.07	(-0.18, 0.10)
Monocular CIVA	0.04 ± 0.08	(-0.08, 0.60)	0.06 ± 0.09	(-0.08, 0.40)	0.05 ± 0.09	(-0.08, 0.30)
Binocular CIVA	0.00 ± 0.05	(-0.08, 0.20)	0.02 ± 0.07	(-0.08, 0.30)	0.02 ± 0.07	(-0.08, 0.22)
Monocular UIVA	0.04 ± 0.08	(-0.08, 0.60)	0.06 ± 0.11	(-0.08, 0.60)	0.06 ± 0.10	(-0.18, 0.30)
Binocular UIVA	0.02 ± 0.07	(-0.08, 0.20)	0.04 ± 0.10	(-0.08, 0.60)	0.03 ± 0.09	(-0.08, 0.30)
Monocular CNVA	0.05 ± 0.10	(-0.08, 0.70)	0.05 ± 0.11	(-0.08, 0.49)	0.05 ± 0.09	(-0.08, 0.30)
Binocular CNVA	0.01 ± 0.10	(-0.08, 0.30)	0.02 ± 0.09	(-0.08, 0.40)	0.01 ± 0.07	(-0.08, 0.20)
Monocular UNVA	0.06 ± 0.10	(-0.08, 0.70)	0.05 ± 0.11	(-0.08, 0.60)	0.05 ± 0.09	(-0.08, 0.30)
Binocular UNVA	0.02 ± 0.08	(-0.08, 0.30)	0.03 ± 0.10	(-0.08, 0.40)	0.03 ± 0.09	(-0.08, 0.30)

*Abbreviations: CDVA* Corrected distance visual acuity, *UDVA* Uncorrected distance visual acuity, *CIVA* Corrected intermediate visual acuity, *UIVA* Uncorrected intermediate visual acuity, *CNVA* Corrected near visual acuity, *UNVA* Uncorrected near visual acuity

**Fig. 2:**
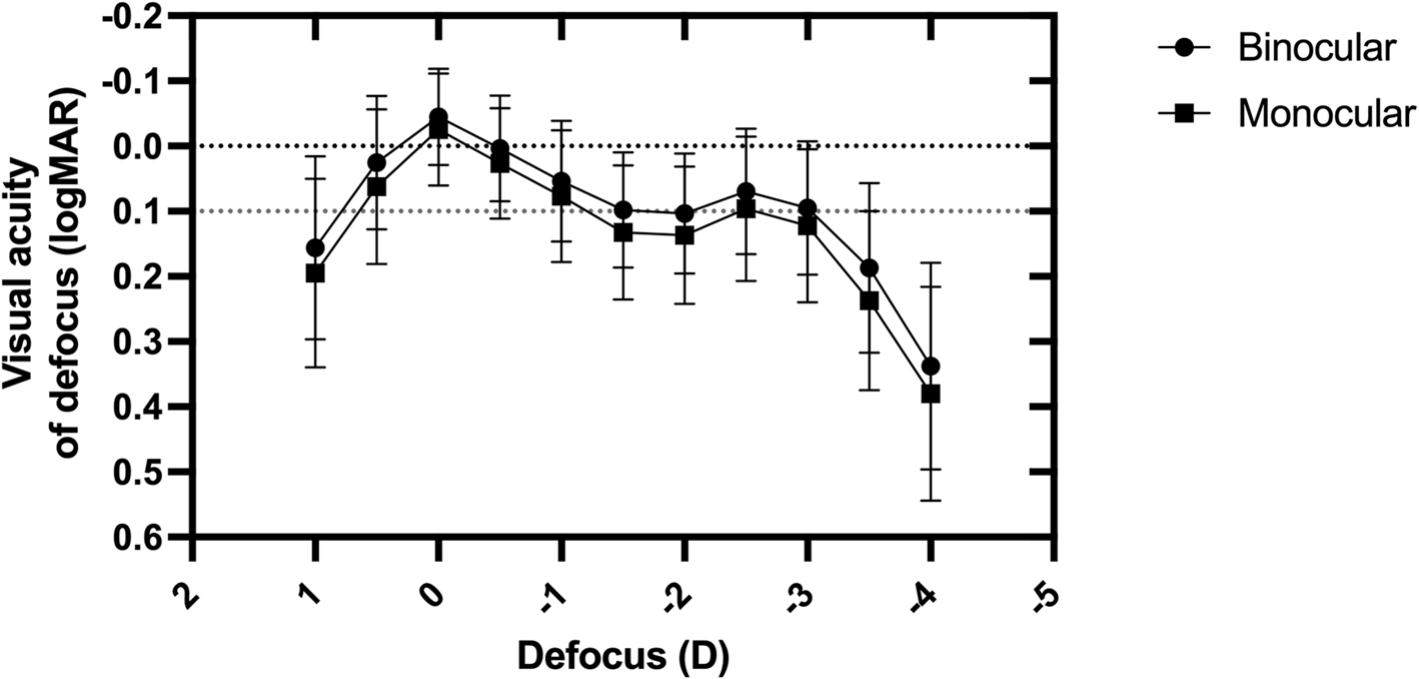
Monocular and binocular defocus curve 6 months postoperatively

The defocus curve showed peak visual acuity at 0.0D for both monocular and binocular measurements (Fig. 2). The VA slightly decreased at the -1.5D intermediate focus before peaking again at the -2.5D near focus. The binocular VA remained at 0.1logMAR or better, and the monocular VA was maintained at 0.15logMAR or better across the range of + 0.5D to -3.0D.

### Refractive outcomes

**Fig. 3:**
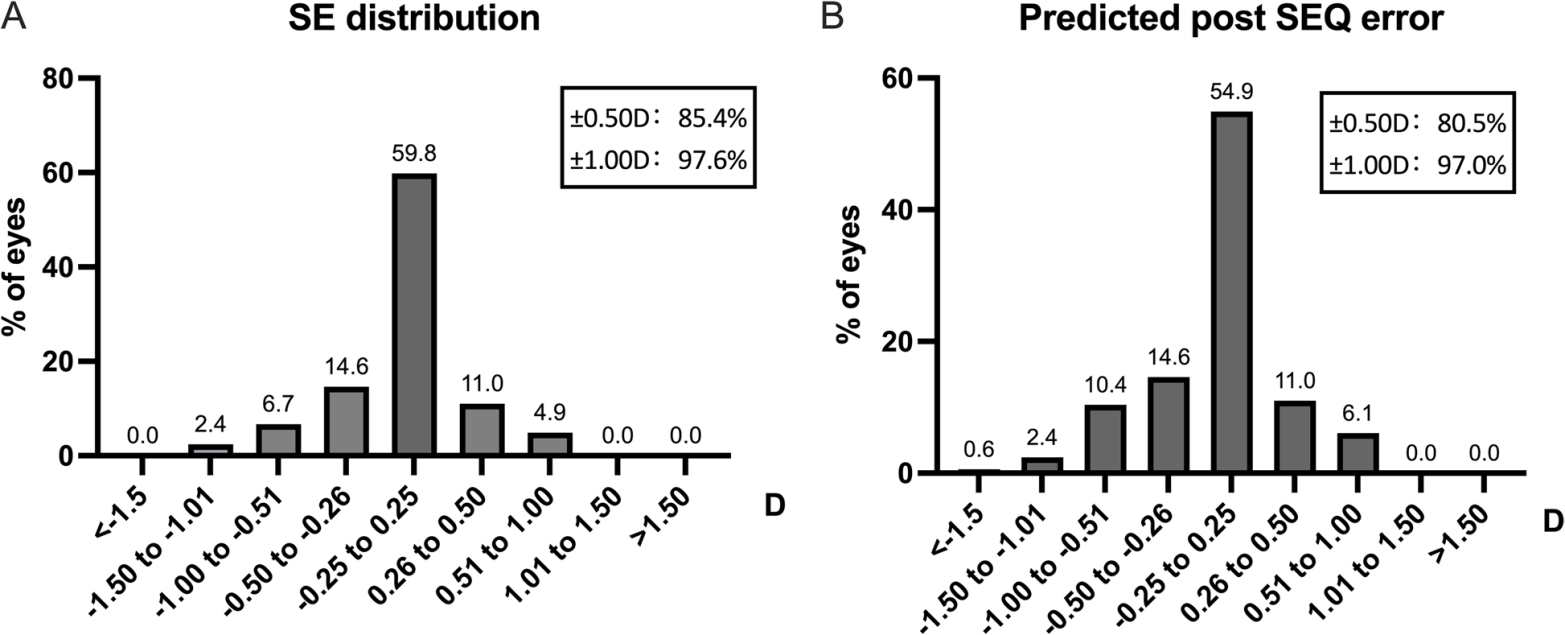
Spherical equivalent 6 months postoperatively (**a**). Predicted postoperative spherical equivalent error 6 months postoperatively (**b**)

Table 3 showed the preoperative and postoperative refractive outcomes. The mean spherical equivalent at 6 months post-surgery was -0.08 ± 0.39 D. 97.6% and 85.4% of eyes were within ± 1.00D and ± 0.50D of emmetropia (Fig. 3 a).

**Table 3:** Preoperative and postoperative refractive outcomes

Refraction (D)	Pre-operation		1 day postoperative		1 month postoperative		6 months postoperative		*P*
	**mean ± SD**	**range**	**mean ± SD**	**range**	**mean ± SD**	**range**	**mean ± SD**	**range**	
Sphere	-1.76 ± 4.37	(-20.00, 7.50)	0.03 ± 0.34	(-0.75, 1.00)	0.10 ± 0.36	(-0.50, 1.25)	0.11 ± 0.37	(-1.00, 1.00)	< 0.001
Cylinder	-0.37 ± 0.55	(-1.75, 1.00)	-0.44 ± 0.41	(-2.25, 0.75)	-0.42 ± 0.36	(-1.50, 0.25)	-0.39 ± 0.40	(-1.50, 0.75)	0.650
SE	-1.89 ± 4.40	(-20.00, 7.50)	-0.18 ± 0.35	(-1.00, 0.63)	-0.13 ± 0.34	(-0.88, 0.75)	-0.08 ± 0.39	(-1.25, 0.88)	< 0.001

*P* Preoperative vs. 6 months postoperative

*SE* Spherical equivalent, *D* Diopter

### Predictability

The predicted postoperative spherical equivalent (SEQ) error was defined as the difference between postoperative SE and predicted postoperative SE. The mean predicted postoperative SEQ error was 0.09 ± 0.40D. 97.0% and 80.5% of eyes had predicted postoperative SEQ error within ± 1.00D and ± 0.50D (Fig. 3 b).

### Objective optical quality and ocular aberrations

Six months post-surgery, significant enhancements were observed in the MTF of corneal, intraocular, and overall ocular measures when compared to preoperative values (*p* < 0.05 for all). Similarly, postoperative Strehl ratios for intraocular and total eye assessments showed a marked increase (*p* < 0.05). Table 4 presents the mean preoperative and postoperative (at 6 months) values for corneal, intraocular, and total eye aberrations. Higher-order aberrations (HOAs), including coma, spherical, and trefoil aberrations, were substantially reduced at 6 months post-surgery (*p* < 0.05). There was also a significant decrease in lower-order aberrations for corneal, intraocular, and total eye measurements (*p* < 0.05). Notably, higher-order aberrations of the total eye at the 6-month follow-up did not exhibit a correlation with visual disturbance phenomena such as halo, glare, starburst, and diplopia (*p* > 0.05 for all).

**Table 4:** Preoperative and postoperative corneal, internal, and ocular wavefront aberrations

		**Preoperative**	**6 months** **Postoperative**	***P***
**Corneal**	MTF	0.427 ± 0.106	0.455 ± 0.120	0.036
	SR	0.211 ± 0.126	0.238 ± 0.170	0.151
	Total aberrations(μm)	0.190 ± 0.075	0.167 ± 0.076	0.007
	LO total(μm)	0.176 ± 0.080	0.155 ± 0.076	0.003
	HO total(μm)	0.064 ± 0.023	0.058 ± 0.031	0.012
	coma(μm)	0.037 ± 0.021	0.032 ± 0.021	0.054
	Spherical(μm)	0.017 ± 0.009	0.015 ± 0.011	0.020
	Trefoil(μm)	0.044 ± 0.020	0.037 ± 0.024	0.001
**Intraocular aberrations**	MTF	0.134 ± 0.115	0.345 ± 0.111	< 0.001
	SR	0.033 ± 0.062	0.196 ± 0.166	< 0.001
	Total aberrations(μm)	1.233 ± 0.945	0.229 ± 0.297	< 0.001
	LO total(μm)	1.195 ± 0.927	0.195 ± 0.197	< 0.001
	HO total(μm)	0.147 ± 0.239	0.104 ± 0.231	< 0.001
	coma(μm)	0.081 ± 0.178	0.048 ± 0.078	< 0.001
	Spherical(μm)	0.008 ± 0.036	-0.016 ± 0.022	< 0.001
	Trefoil(μm)	0.073 ± 0.099	0.055 ± 0.147	< 0.001
**Total eye**	MTF	0.145 ± 0.125	0.339 ± 0.097	< 0.001
	SR	0.037 ± 0.068	0.146 ± 0.105	< 0.001
	Total aberrations(μm)	1.217 ± 0.979	0.259 ± 0.298	< 0.001
	LO total(μm)	1.219 ± 0.979	0.226 ± 0.199	< 0.001
	HO total(μm)	0.156 ± 0.239	0.106 ± 0.232	< 0.001
	coma(μm)	0.091 ± 0.179	0.052 ± 0.079	< 0.001
	Spherical(μm)	0.025 ± 0.038	-0.001 ± 0.022	< 0.001
	Trefoil(μm)	0.072 ± 0.100	0.057 ± 0.149	0.001

*Abbreviations*:* MTF* Modulation transfer function, *SR* Strehl ratio, *LO* Low order, *HO* High order

### Patient-reported satisfaction

**Fig. 4:**
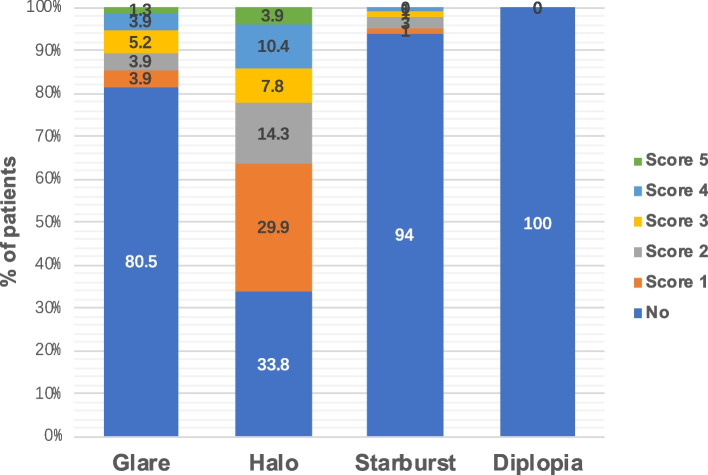
Patient-reported visual phenomenon at 6 months postoperatively

A total of 77 VF-14 questionnaires were returned. Table 5 outlines the VF-14 items along with the patient-reported scores for each. The mean VF-14 score was 94.3 ± 10.2, with tasks such as reading small print, engaging in handwork, and driving at night scoring below 90 points on average. In contrast, scores for other VF-14 items generally exceeded 90 points. A substantial 93.5% of patients (72 individuals) reported no dependence on spectacles post-surgery. A minority of patients reported needing glasses for distance (2.6%) or near activities (3.9%). Optical phenomena were commonly reported, with halos being the most prevalent (66.2%), followed by glare (18.2%), and starburst (7.8%) as illustrated in Fig. 4. A significant majority, 92.2% (71 patients), expressed satisfaction with the surgical outcome, and 94.8% (73 patients) would recommend the procedure to friends and family.

**Table 5:** VF-14 questionnaire outcomes

Question	Average score	No difficulty (n, %)	A little difficult (n, %)	Moderate difficulty (n, %)	A great deal of difficult (n, %)	Unable to do (n, %)	Not applicable (n, %)
Do you have any difficulty, even with glasses, reading small print, such as labels on medicine bottles, a telephone book, or food labels?	81.5 ± 19.6	32(41.6%)	37(48.1%)	4(5.2%)	4(5.2%)	0(0.0%)	0(0.0%)
Do you have any difficulty, even with glasses, reading a newspaper or a book?	92.9 ± 16.7	62(80.5%)	10(13.0%)	3(3.9%)	2(2.6%)	0(0.0%)	0(0.0%)
Do you have any difficulty, even with glasses, reading a large print book or, newspaper or numbers on a telephone?	98.4 ± 10.2	75(97.4%)	0(0.0%)	1(1.3%)	1(1.3%)	0(0.0%)	0(0.0%)
Do you have any difficulty, even with glasses, recognizing people when they are close to you?	98.1 ± 10.5	74(96.1%)	1(1.3%)	1(1.3%)	1(1.3%)	0(0.0%)	0(0.0%)
Do you have any difficulty, even with glasses, seeing steps, stairs or curbs?	98.1 ± 12.7	75(97.4%)	0(0.0%)	1(1.3%)	0(0.0%)	1(1.3%)	0(0.0%)
Do you have any difficulty, even with glasses, reading traffic signs, street signs, or store signs?	96.4 ± 13.9	71(92.2%)	3(3.9%)	1(1.3%)	2(2.6%)	0(0.0%)	0(0.0%)
Do you have any difficulty, even with glasses, doing fine handwork like sewing, knitting, crocheting, or carpentry?	85.5 ± 22.8	47(61.0%)	22(28.6%)	4(5.2%)	2(2.6%)	2(2.6%)	0(0.0%)
Do you have any difficulty, even with glasses, writing checks or filling out forms?	97.1 ± 13.4	72(93.5%)	1(1.3%)	1(1.3%)	2(2.6%)	0(0.0%)	1(1.3%)
Do you have any difficulty, even with glasses, playing games such as mahjong, card games, and chess?	98.1 ± 10.5	74(96.1%)	1(1.3%)	1(1.3%)	1(1.3%)	0(0.0%)	0(0.0%)
Do you have any difficulty, even with glasses, taking part in sports like badminton, gate ball, table tennis, basketball, walking, doing exercises, tai chi?	97.4 ± 11.2	72(93.5%)	3(3.9%)	1(1.3%)	1(1.3%)	0(0.0%)	0(0.0%)
Do you have any difficulty, even with glasses, cooking?	98.7 ± 8.0	75(97.4%)	0(0.0%)	2(2.6%)	0(0.0%)	0(0.0%)	0(0.0%)
Do you have any difficulty, even with glasses, watching television?	97.4 ± 13.4	74(96.1%)	1(1.3%)	0(0.0%)	1(1.3%)	1(1.3%)	0(0.0%)
How much difficulty do you have driving during the day because of your vision?	97.1 ± 13.4	72(93.5%)	3(3.9%)	1(1.3%)	0(0.0%)	1(1.3%)	0(0.0%)
How much difficulty do you have driving at night because of your vision?	83.4 ± 21.7	39(50.6%)	30(39.0%)	5(6.5%)	1(1.3%)	2(2.6%)	0(0.0%)

Patients are further divided into myopia (SE < –0.5 D), emmetropia (–0.5 D ≤ SE ≤ 0.5 D), and hyperopia (SE > 0.5 D) groups based on preoperative spherical equivalent. The mean VF-14 scores were 94.0 ± 10.2, 94.2 ± 7.1, and 94.8 ± 12.7 for myopia, emmetropia, and hyperopia groups (*P* = 0.205). For each item of the questionnaire, there was no significant difference in the scores between the three groups (*P* > 0.05 for all items). Patient satisfaction rates were similar in all three groups. Halos were more frequently reported in the emmetropia group (80.0%) compared with myopia group (50.0%; *P* = 0.007) and hyperopia group (78.3%; *P* = 0.052). Glare and starburst appeared in similar rates in each group (Table 6).

**Table 6:** Patient satisfaction and visual phenomenon in myopia, emmetropia, and hyperopia groups

	**Myopia**	**Emmetropia**	**Hyperopia**	P*	P^**^	P^***^
VF-14 score	94.0 ± 10.2	94.2 ± 7.1	94.8 ± 12.7	> 0.999	0.732	0.236
Satisfaction rate (%)	94.10%	90%	91.30%	0.622	> 0.999	> 0.999
Glare (%)	17.60%	10.00%	26.10%	0.695	0.517	0.250
Halo (%)	50.00%	80.00%	78.30%	0.007	0.052	0.438
Starburst (%)	11.80%	10.00%	0.00%	> 0.999	0.140	0.210
Diplopia (%)	0	0	0	-	-	-

P^*^ comparison between myopia group and emmetropia group

P^**^ comparison between myopia group and hyperopia group

P^***^ comparison between emmetropia group and hyperopia group

### Safety

The surgical interventions were free of intraoperative complications and did not necessitate any IOL exchanges. Posterior capsule opacification was noted in 153 eyes (83.2%), with Nd: YAG laser treatment performed six months post-surgery. Cystoid macular edema (CME) occurred in four eyes from two patients (2.3%) two months post-surgery, resolving after four weeks of nonsteroidal anti-inflammatory eye drop treatment. Dry eye symptoms were reported by 7 patients (8.5%), affecting 14 eyes, and were managed with artificial tears.

## Discussion

The preference for RLE using trifocal IOLs for spectacle independence is increasing and it is a challenge for ophthalmologists. Yet, focused studies on RLE in presbyopic but non-cataractous individuals are limited. Such patients may have unique perceptions of surgical outcomes compared to those with cataracts, given the differences in procedural expectations and sensitivity to postoperative visual disturbances. Consequently, comprehensive evaluation of these patients' experiences is essential for determining RLE's therapeutic efficacy. In our study, patients visited the clinic seeking opportunities to achieve spectacle independence. They were informed about various treatment options, including spectacles, laser refractive correction, ICL, and RLE, along with the pros and cons of each procedure. The patients were fully aware of the benefits and limitations of the procedure, including likelihood of photic phenomena, possible need of YAG laser treatment, and possible need for spectacles postoperatively. Both the doctor and the patient made the decision with caution based on reasonable expectations. Our study found that bilateral trifocal IOL implantation in presbyopic patients without cataracts significantly improved visual acuity over a range of distances, leading to a notable degree of patient satisfaction.

### RLE patient demographics

RLE and cataract surgery are tailored to differing patient needs. Notably, cataracts affect a mere 3.9% of individuals between the ages of 55 to 64 [14]. Patients opting for RLE are younger, around 53 to 59 years old, compared to those undergoing cataract surgery [11, 12, 15]. The average age of patients undergone RLE in our study was 54.8 ± 6.0 years. These patients are professionally active in China and have substantial visual demands across varying distances, both in daily life and at work. Near vision difficulties can significantly impair these active individuals' work performance and life quality [16, 17]. Therefore, patients exhibit a pronounced inclination to enhance their near vision to fulfill everyday visual requirements.

### DLI

The DLI, an innovative objective metric developed by the iTrace Visual Function Analyzer, quantifies the degradation of the lens. DLI reflects lens health and performance [15] with research by Li and colleagues proposing a DLI threshold of ≤ 5.7 as a criterion for cataract surgery [18]. Recent research by Martínez-Plaza has reported on DLI values in a healthy cohort, noting mean DLIs of 8.89 ± 2.00 in individuals under 50 and 6.71 ± 2.97 in those 50 and older [19]. Additionally, Kaweri et al. identified a DLI of 7.810 ± 0.168 in a presbyopic population [15]. Our study's DLI average of 7.71 ± 2.53 aligns with those reported in non-cataractous subjects by prior studies, affirming our cohort's non-cataractous status.

### IOL power calculation

In our study, the Barrett Universal II TK formula was utilized for IOL power calculation. At 6 months postoperative, the mean spherical equivalent (SE) was 0.11 ± 0.37, with 80.5% of eyes within ± 0.50D of the targeted refractive outcome. These results in our Chinese cohort are in agreement with those reported in a similar recent study involving the same trifocal IOL, where 80.9% of Spanish presbyopic patients' eyes were within ± 0.5 D of the anticipated refractive target [20].

### Objective optical quality and ocular aberrations

Aberrations, MTF, and SR serve as indicators of objective visual quality. At a six-month postoperative interval, we observed significant improvements in MTF and SR, denoting enhanced visual precision. Notably, intraocular aberrations, mainly lens-derived, diminished considerably. In comparison with a prior study by Zhang et al., which reported total intraocular HOAs of 0.212 μm in right eyes and 0.166 μm in left eyes in healthy phakic individuals [21], our preoperative HOA measurements were lower, likely due to the smaller pupil zone assessed (3 mm in our study versus 4 mm in Zhang’s). The substantial reduction in both intraocular and overall eye HOAs post-surgery indicates an enhancement in visual quality, with the potential to mitigate symptoms associated with visual disturbances.

### Patient-reported satisfaction

Photic phenomena have been reported to affect patient satisfaction after trifocal IOL implantation [22, 23]. In our research, halo was the most commonly encountered photic phenomenon, affecting 66.2% of patients. These findings are consistent with Mendicute et al., who observed that around 80% of patients perceived halos after surgery [24]. We also found that emmetropic patient before surgery tend to have higher incidence of experiencing halo postoperatively. Emmetropic patients are likely to have better visual quality compared with those have refractive errors. One possible explanation for this result is emmetropic patients are more sensitive to photic phenomena, however further studies are needed to better evaluate photic phenomena for patients with different ocular conditions. Despite of the presence of photic phenomena, patients who have undergone RLE still have a high level of satisfaction, considering these optical discomforts to be acceptable.

The VF-14 questionnaire outcomes from our cohort after trifocal intraocular lens implantation reflect substantial satisfaction in performing daily visual tasks. Night driving was highlighted as problematic for some, presumably due to nighttime aberrations like halos and glare. Though most patients find this discomfort acceptable, it is important for doctors to concern when a patient consults for this procedure. For patients who needs to drive at night a lot, the doctor should be extremely cautious because the surgery could result in dissatisfaction. These findings align with those from other studies [20, 25, 26]. Despite such challenges, an overwhelming majority (92%) reported satisfaction with their surgical outcome, with 93% achieving complete independence from spectacles.

### PCO and treatment

As emphasized by patient feedback, those with presbyopia but without cataracts often have considerable expectations for their vision post-surgery. Any thickening or loss of transparency in the posterior capsule can lead to visual disruptions. Previous research has noted Nd: YAG capsulotomy rates varying from 11.1% to 42.7%, with an upward trend correlating with longer follow-up times [20, 27–29]. Our study presents a higher Nd: YAG capsulotomy rate of 83.2%, attributable to an earlier intervention with Nd: YAG capsulotomy with a high standard as mentioned above. Early Nd: YAG capsulotomy did not result in any serious complications throughout the follow-up period. Further extensive research is warranted to ascertain if early Nd: YAG capsulotomy confers advantages to patients receiving trifocal IOLs.

### Safety

Our study has addressed specific concerns about the safety of the surgery. Presbyopic patients are younger and have better baseline visual acuity compared with cataract patients, so complications should be avoided. This surgical procedure demands a high level of precision and ought to be executed by highly skilled surgeons. Detailed patient guidance and communication about possible postoperative visual phenomena should be addressed so that patients can have reasonable expectations about the surgery. Our study has proved the safety of the surgery, but additional studies with longer follow-ups would be necessary to evaluate the long-term safety of the surgery.

### Limitation

The study is a retrospective non-interventional study with a limited sample size. Another limitation of the current study is the lack of contrast sensitivity evaluation. Previous studies have thoroughly reported good contrast sensitivity to different spatial frequencies, especially medium spatial frequencies, after implantation of this trifocal IOL [27, 30]. The characteristics of contrast sensitivity are related to the optic resign of the IOL. Other than contrast sensitivity evaluation, we evaluated other objective measurements such as aberrations, and we also evaluated subjective visual experience with the VF-14 questionnaire with additional questions about visual phenomena.

## Conclusions

To summarize, our findings affirm the safety and efficacy of refractive lens exchange using the AT LISA tri 839MP IOL within the Chinese presbyopic demographic. The study underscores the trifocal IOL's role in bolstering visual performance across multiple distances, contributing to substantial patient satisfaction post-surgery. A detailed assessment encompassing visual acuity, refractive accuracy, objective optical quality, and subjective patient-reported experiences substantiates the potential for achieving spectacle independence through this intervention. Careful patient selection and meticulous preoperative discussions to align expectations, including the possibility of photic phenomena, are crucial for maximizing patient contentment with the outcomes.

## Data Availability

The datasets presented in this study is available from the corresponding author upon reasonable request.

## Abbreviations

RLE: Refractive lens exchange
IOL: Intraocular lens
BCVA: Best-corrected visual acuity
UDVA: Uncorrected distance visual acuity
UIVA: Uncorrected intermediate visual acuity
UNVA: Uncorrected near visual acuity
CDVA: Corrected distance visual acuity
CIVA: Corrected intermediate visual acuity
CNVA: Corrected near visual acuity
OCT: Optical coherence tomography
DLI: Dysfunctional lens index
MTF: Modulation transfer function
SR: Strehl ratio
RMS: Root mean square
D: Dioptor
CCC: Continuous curvilinear capsulorhexis
VF-14: Visual function-14
PCO: Posterior capsule opacification
Nd: YAG, Neodymium-doped yttrium aluminum garnet
SEQ: Spherical equivalent
HOA: Higher-order aberration
CME: Cystoid macular edema
